# Monitoring Radiographic Brain Tumor Progression

**DOI:** 10.3390/toxins3030191

**Published:** 2011-03-15

**Authors:** Ankit I. Mehta, Charles W. Kanaly, Allan H. Friedman, Darell D. Bigner, John H. Sampson

**Affiliations:** Division of Neurosurgery, Duke University Medical Center, Box 3807, Durham, NC 27710, USA; Email: charles.kanaly@duke.edu (C.W.K.); allan.friedman@duke.edu (A.H.F.); darell.bigner@duke.edu (D.D.B.); john.sampson@duke.edu (J.H.S.)

**Keywords:** glioblastoma, radiographic progression, tumor progression, MRI

## Abstract

Determining radiographic progression in primary malignant brain tumors has posed a significant challenge to the neuroncology community. Glioblastoma multiforme (GBM, WHO Grade IV) through its inherent heterogeneous enhancement, growth patterns, and irregular nature has been difficult to assess for progression. Our ability to detect tumor progression radiographically remains inadequate. Despite the advanced imaging techniques, detecting tumor progression continues to be a clinical challenge. Here we review the different criteria used to detect tumor progression, and highlight the inherent challenges with detection of progression.

## 1. Introduction

Determining radiographic progression in primary malignant brain tumors has posed a significant challenge to the neuroncology community. Glioblastoma multiforme (GBM, WHO Grade IV), through its inherent heterogeneous enhancement, growth patterns, and irregular nature, has been difficult to assess for progression. With new immunotherapeutic therapies, stereotactic radiosurgery, anti-VEGF inhibitors, and chemotherapeutics providing efficacy in GBM, it has become more critical for the neuroncology community to quickly and accurately determine tumor progression [1,2]. Despite these advances in therapeutics, our ability to detect tumor progression radiographically remains inadequate. Despite the advanced imaging techniques, detecting tumor progression continues to be a clinical challenge. Here we review the different criteria used to detect tumor progression, and highlight the inherent challenges with detection of progression. 

## 2. MRI Imaging of Enhancement

After a GBM surgical resection, patients receive serial MRI scans to assess for changes in enhancement. An MRI scan should be performed within 48 hours of surgical resection to assess baseline enhancement and assess for postoperative infarcts. Unlike CT scans that have a straight linear correlation between iodine concentration and Hounsfield units of enhancement, MRI has an inherent difficultly in determining a cutoff to judge enhancement. Enhancement on MRI scan and tissue concentration of Gd-DTPA have a complex relationship, that is bimodal in nature with an initial increased enhancement in signal intensity with contrast and then a decrease after a point [3]. The enhancement seen on MRI is not restricted to the tumor itself but includes the vasculature, parenchyma, and nasal mucosa. Blood products in the tumor may also produce signal changes similar to enhancing tissue. These challenges make quantification of enhancement and changes in tumor appearance more difficult to appreciate by the neuroncology community.

## 3. Pseudoprogression/Radionecrosis/Pseudoresponse

When changes in enhancement are detected on MRI, there is difficulty in determining if there is true tumor recurrence, radiation necrosis, or pseudoprogression most often associated with Temozolomide treatment [4]. Pseudoprogression is a novel but well documented phenomena where new enhancement of tissue is detected usually within three months of radiation, but with necrosis or gliosis noted on biopsy. The incidence of pseudoprogression has been considerable with 32 out of 103 patients or 31.1% of GBM patients recently reported [5]. Despite being a well documented phenomenon, the mechanism behind pseudoprogression continues to be poorly understood. The current theory is that chemotherapy and radiotherapy induce tumor and endothelial cell death creating secondary edema and increased vessel permeability localized to the tumor area [6]. 

Conversely, radionecrosis can occur 3–12 months after radiotherapy [7]. Radionecrosis occurs where there is local tissue reaction after radiotherapy with signs of a disrupted blood-brain barrier and edema. Histopathology demonstrates gliosis, endothelial thickening, necrosis, edema and thrombosis [6].

Tumor progression, when assessed by measurement of tissue enhancement, will therefore not accurately quantify tumor burden if there is a significant amount of non-enhancing tumor. Even in enhancing tumors, there has recently been increasing use of anti-angiogenic agents such as bevacizumab that normalize vasculature and decrease enhancement leading to potential under- and over-interpretations [8]. Other authors have noted that since enhancement can change due to radiation necrosis, pseudoprogression, steroid treatment, or pseudoresponse, it is not always the case that enhancement reflects changes in the underlying tumor [9]. Difficulty visualizing non-enhancing tumor burden is a problem for most proposed methods of assessing tumor response, and some authors have proposed that response criteria in these situations may have to be altered to include both radiologic changes and measurements of circulating biomarkers [10]. 

## 4. Current Methods of Assessing Tumor Progression

### 4.1. Macdonald Criteria

The MacDonald criteria for determining tumor progression is determined through assessing the increase in size of an enhancing tumor on consecutive MRI scans and clinical assessment. Treatment responses are divided into four categories: complete response, partial response, progressive disease, and stable disease. Complete response occurs when there is a disappearance of all enhancing tumor on consecutive MRI scans at least one month apart, off steroids, and the patient is neurologically stable or improved. Partial response occurs at a >50% reduction in size of enhancing tumor on consecutive MRI scans at least one month apart, steroids stable or reduced, and neurologically stable or improved. Progressive disease occurs when there is a >25% increase in size of enhancing tumor on consecutive MRI scans, patient is neurologically worse, and steroids stable or increased. Stable disease occurs in all remaining situations. The size of a tumor’s largest cross sectional area is used to assess for changes [4]. 

There are inherent difficulties in the MacDonald criteria when used rigorously to determine tumor progression. The criteria cannot be applied to all clinical criteria especially when the contrast enhancing region does not encompass all biologically and clinically active disease. MRI enhancement is not specific towards glioma progression when inflammatory response, breakdown of the blood brain barrier, and other pathological responses demonstrate enhancing regions [11]. In addition, there is a great deal of interobserver variability when using the McDonald criteria to assess progression, with Vos *et al*. reporting a 65% and 55% correspondence of progression on CT and MRI, respectively [12]. 

### 4.2. RECIST Criteria

**Figure 1:**
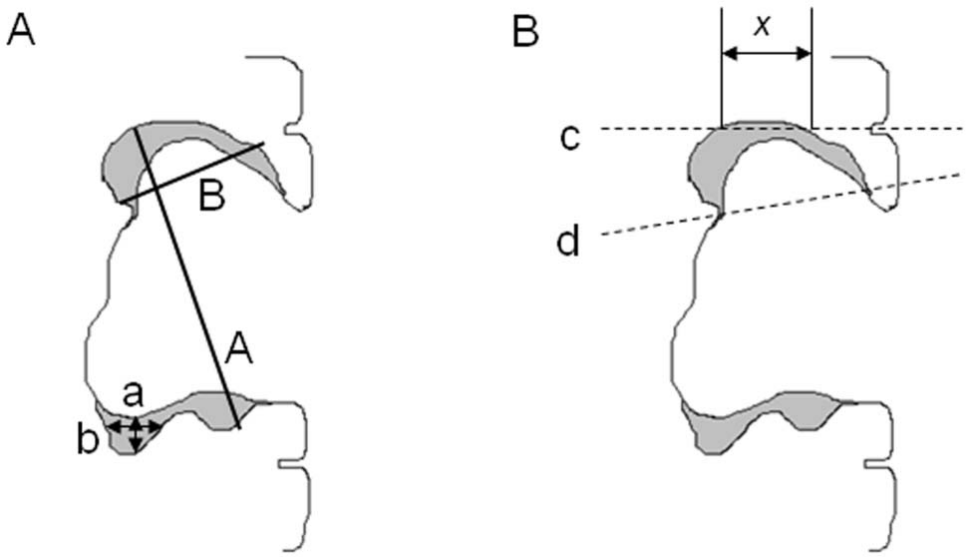
Traditional Non-Volumetric Measurements do not Adequately Describe Residual Enhancement in Surgical Resection Cavities. (**A**) This schematic resection cavity has residual rim enhancement in gray. RECIST criteria measurement ‘A’ or ‘a’ or ‘b’ or Macdonald criteria measurement ‘A*B’ or ‘a*b’ would not adequately describe residual tumor volume and additional tumor growth around the rim or collapse of the resection cavity may be over- or under-interpreted. (**B**) Differences in axial slice acquisition also impact measurements made by traditional criteria more than volumetric measurements. One scan could obtain axial slice ‘c’ with enhancing tumor measurement ‘x’ but a subsequent scan in the same patient could obtain axial slice ‘d’, causing an incorrect assessment of tumor response. (Reprinted with permission from PLOSOne [15]).

The RECIST Criteria, Response Evaluation Criteria in Solid Tumors, also classifies patients into four groups on repeat imaging. Complete response occurs with disappearance of all lesions with confirmation at four weeks. Partial response is a >30% decrease in the sum of the longest diameters of all lesions with confirmation at four weeks. Stable disease is a decrease of less than 30% or an increase of less than 20% in the sum of the longest diameters of all lesions. Progressive disease is an >20% increase in the sum of the longest diameter of all lesions (Figure 1) [13]. 

The pitfalls with the RECIST criteria include the difficulty of measuring cystic GBM lesions that do not encompass the entire diameter of enhancement. In addition, GBM enhancement occurs as a rim enhancement phenomenon where a considerable variability would exist in determining the diameter of tumor.

### 4.3. WHO Criteria

The WHO developed a response criteria on MRI images that used the sum of the estimated areas of all lesions based on the longest diameter by the greatest perpendicular diameter. Patients were classified into four groups. Complete response is defined as disappearance of all lesions as determined by two MRIs at least four weeks apart. Partial response is ≥50% decrease in the sum of total tumor area by two MRIs at least four weeks apart. No change, which is equivalent to stable disease, is defined as less than a 50% decrease or less than a 25% increase in the sum of total tumor area. Progressive disease is defined as ≥25% increase in the size of one or more lesions or the appearance of new lesions [14]. The WHO criteria has similar shortcomings as the RECIST criteria with the subjective nature of measuring tumor burden in the resection cavity, cystic tumor lesions, and is unclear about daughter lesions. 

### 4.4. RANO Criteria

The RANO response criteria were developed with the inherent pitfalls of conventional criteria assessing for only enhancing tumors. The inherent pitfalls of the conventional criteria particularly entail pseudoprogression from radiochemotherapy, lack of steroid factoring into response, and non-enhancing tumor with anti-angiogenic therapies [16]. The RANO criteria develop MRI imaging characteristics to assess responses as follows.

#### 4.4.1. Complete Response

Disappearance of all enhancing and non-measurable disease for a period of at least four weeks. Stable or improved nonenhancing lesions on T2/FLAIR lesions on same or lower dosage of steroids from baseline [16].

#### 4.4.2. Partial Response

Greater than 50% decrease of enhancing tumor in diameter sustained for four weeks; no progression of non-measurable disease; no new lesions; and stable or improved T2/FLAIR lesions on same or lower dosage of steroids from baseline [16].

#### 4.4.3. Stable Disease

Stable T2/FLAIR lesions on same or lower dosage of steroids from baseline and clinically stable status [16]. 

#### 4.4.4. Progression

The following criteria are met: greater than or equal to 25% increase in the sum of diameters of enhancing lesions on stable or increased steroid dosage, a significant increase in T2/FLAIR nonenhancing lesions, appearance of new lesions, or clear clinical deterioration [16]. 

## 5. Challenges and Future of Tumor Progression Imaging

Currently the standard of therapy for detecting tumor progression has been using the MacDonald RECIST, or WHO Criteria with contrast enhancing MRI studies. From this report, the accuracy of this methodology has significant interobserver variability, difficulty in interpretation, and poor specificity between radiation necrosis and tumor progression. However due to the availability of MRI scanners and the frequency of MRI scans in these patients, MRI progression has been predominantly used for clinical protocols to detect progression. 

The authors feel that MRI analysis needs to be improved to reduce the subjective calculation of diameters and enhancing volume on the scans. We recommend using volumetric software to assess changes in enhancing tumor volumes. This has inherent difficulties with variable enhancement levels and other areas of enhancement that include the nasal mucosa and blood products. Further investigation in volumetric changes needs to verified and utilized prospectively to detect tumor progression. 

Volumetric methods demonstrate promise in the response assessment of enhancing brain tumors. Such methods can account for enhancing tumor despite expected postoperative collapse of the resection cavity. Despite this detection of enhancing brain volumes, there is an inherent difficulty in assessing residual enhancement from blood in the resection cavity. These situations frequently occur in brain tumor patients after surgical resection and have been difficult to describe with traditional response assessment methods.

Technical differences, such as contrast dose and gantry angle, for serial MRI scans can lead to the false appearance of changes in the amount of tumor enhancement. A cutoff for enhancement that is calculated from each scan can help minimize the variations in contrast bolus between different MRI scans. This increases the universability of a program that analyzes different MRI scans. 

Reproducibility continues to be a pitfall in calculating tumor volumes [12]. Vos *et al*. investigated this interobserver variability by assessing radiographic response criteria for 35 patients with gliomas using five experienced clinicians. The authors found a poor interobserver variability with an intraclass correlation coefficient of 0.51 amongst clinicians assessing radiographic response using McDonald criteria [12]. This pitfall of interobserver variability increases the necessity for automation. In support of this, Schwartz *et al.* found that in CT assessment of solid tumors, techniques that employed increased automation obtained results that were more accurate and consistent than manual methods [17]. Other studies using automated CT volumetric methods in pulmonary tumors suggest superiority when compared to manual RECIST measurements [18,19]. For gliomas, Sorensen *et al.* found that a computer-assisted perimeter method of volume calculation produced less inter- and intra-user variability than a manual volumetric calculation that used diameter measurements [20]. Other semi-automated tumor assessment methods, including automatic segmentation methods that use fuzzy clustering and interactive watershed algorithms, do not take into account tumor enhancement specifically [21,22,23]. This is due to the difficulty of the enhancement criteria of tumor amongst the segmented scans compared to normal parenchyma.

An important difficulty with measuring enhancing brain tumors on MRI is that there is no quantitative cutoff for tissue enhancement on MRI. There have been a few previous descriptions of a formulaic determination of an enhancement threshold based on the initial peak enhancing signal increase, but this has not been widely accepted [24,25]. Many previous methods have simply utilized expert opinion to select the enhancing tissue according to their best judgment, however this technique invites significant subjective error, as different experts may have different opinions. Use of a standard threshold has precedence in other fields. With positron emission tomography (PET) scans, the detected intensity tapers off over distance from the source, so it is difficult to delineate precisely where the intensity is no longer apparent. A number of different methods have been attempted to estimate the tumor region of interest, and a set 40% threshold of either the source-to-background (S/B) ratio or of the maximum standardized uptake value (SUV) are commonly advocated techniques [26,27]. 

There are concerns that the measurement of tissue enhancement will not accurately quantify tumor burden if there is a significant amount of non-enhancing tumor. Even in enhancing tumors, there has recently been increasing use of anti-angiogenic agents such as bevacizumab that normalize vasculature and decrease enhancement leading to potential over-interpretations [8,28,29]. Other authors have noted that since enhancement can change due to radiation necrosis, pseudo-progression, steroid treatment, or pseudo-response, enhancement does not always reflect changes in the underlying tumor [9]. The RANO criteria were drafted to attempt to address these limitations [30]. Difficulty visualizing non-enhancing tumor burden is a problem for most proposed methods of assessing tumor response, and some authors have advocated that response criteria in these situations may have to be altered to include both radiologic changes and measurements of circulating biomarkers [10]. Unfortunately, these limitations are equally applicable to the Macdonald or RECIST criteria. The initial paper by Macdonald *et al.* even acknowledges that their criteria should not be applied to non-enhancing tumor [4]. Determination of the magnitude and time course of these different changes may lead to greater ability to distinguish between actual disease recurrence and other causes of enhancing volume change.

Pseudoprogression and radiation necrosis result in enhancement after radiation therapy and Temozolomide in patients with GBM. There is not a standard manner to differentiate these pathologies from tumor progression. The authors believe that a biomarker would be helpful in determining the difference between pseudoprogression and tumor progression. However this is a major pitfall and thus far can only be determined through a stereotactic brain biopsy. 

**Figure 2:**
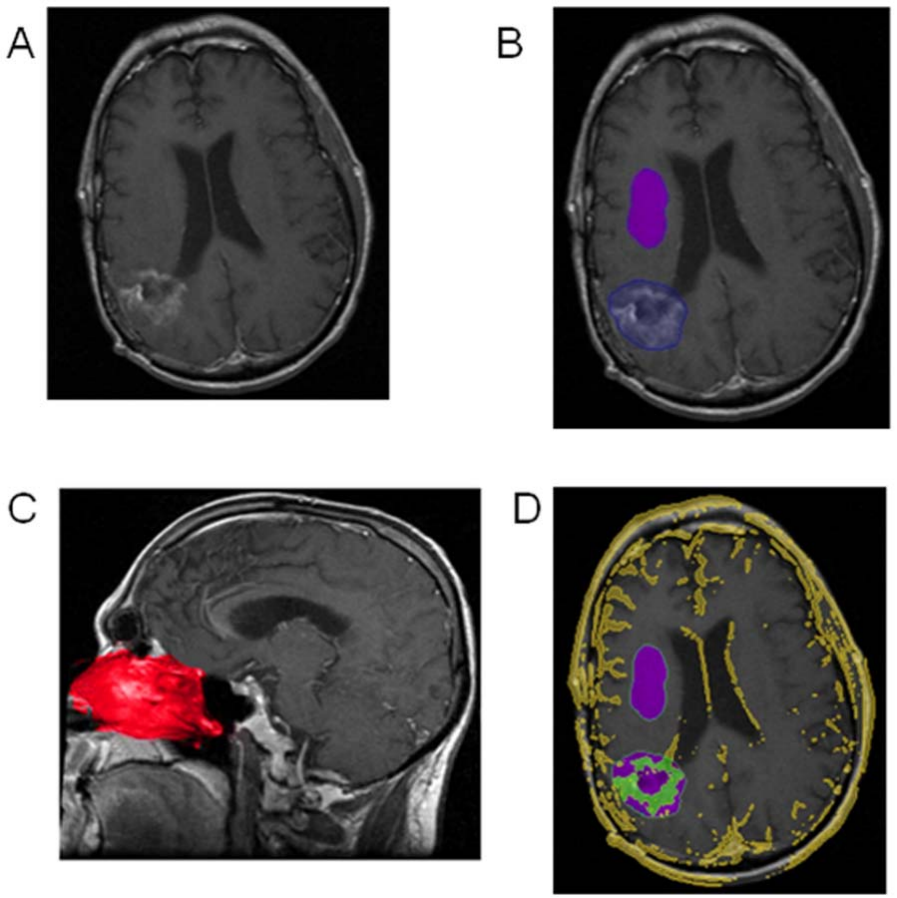
Automated Assessment of Enhancing Tumor Volume. (**A**) T1-weighted post-contrast axial images are automatically fused with the pre-contrast sequences. (**B**) The tumor region of interest (blue area) and nearby normal brain (purple area) are outlined roughly by hand. (**C**) The enhancing nasal mucosa region is automatically detected with a built-in anatomic atlas (red area) and serves as a threshold for enhancement. (**D**) Tissue that is present on the post-contrast images but not the pre-contrast that is above the enhancement threshold appears in yellow. This includes enhancing tissue such as vasculature, tumor, and superficial structures. Enhancing tumor volume is defined as the green area within the manually-defined blue tumor region of interest. (Reprinted with permission from PLOSOne [15]).

In the future, we would suggest that progression should be determined through an automated method determining radiographic characteristics of a tumor such as enhancement. In addition it is important that this tool would provide a high interuser reliability, therefore creating a consistent manner to determine radiographic progression. The authors reported on the novel technology called Velocity that uses volumetric software to determine tumor enhancement volumes (Figure 2) [15]. If there is a standard automated software that determines tumor progression, as a field we can transform a subjective interpretation into an objective analysis.
